# Automatic Measurements of Fetal Lateral Ventricles in 2D Ultrasound Images Using Deep Learning

**DOI:** 10.3389/fneur.2020.00526

**Published:** 2020-07-17

**Authors:** Xijie Chen, Miao He, Tingting Dan, Nan Wang, Meifang Lin, Lihe Zhang, Jianbo Xian, Hongmin Cai, Hongning Xie

**Affiliations:** ^1^School of Computer Science and Engineering, South China University of Technology, Guangzhou, China; ^2^Affiliated Hospital of Sun Yat-sen University, Guangzhou, China; ^3^Guangzhou Aiyunji Information Technology Co., Ltd, Guangzhou, China

**Keywords:** biometric measurement, computer-aided diagnosis, ultrasound, fetal head, deep learning, lateral ventricle

## Abstract

Measurement of the width of fetal lateral ventricles (LVs) in prenatal ultrasound (US) images is essential for antenatal neuronographic assessment. However, the manual measurement of LV width is highly subjective and relies on the clinical experience of scanners. To deal with this challenge, we propose a computer-aided detection framework for automatic measurement of fetal LVs in two-dimensional US images. First, we train a deep convolutional network on 2,400 images of LVs to perform pixel-wise segmentation. Then, the number of pixels per centimeter (PPC), a vital parameter for quantifying the caliper in US images, is obtained via morphological operations guided by prior knowledge. The estimated PPC, upon conversion to a physical length, is used to determine the diameter of the LV by employing the minimum enclosing rectangle method. Extensive experiments on a self-collected dataset demonstrate that the proposed method achieves superior performance over manual measurement, with a mean absolute measurement error of 1.8 mm. The proposed method is fully automatic and is shown to be capable of reducing measurement bias caused by improper US scanning.

## 1. Introduction

**Figure 1:**
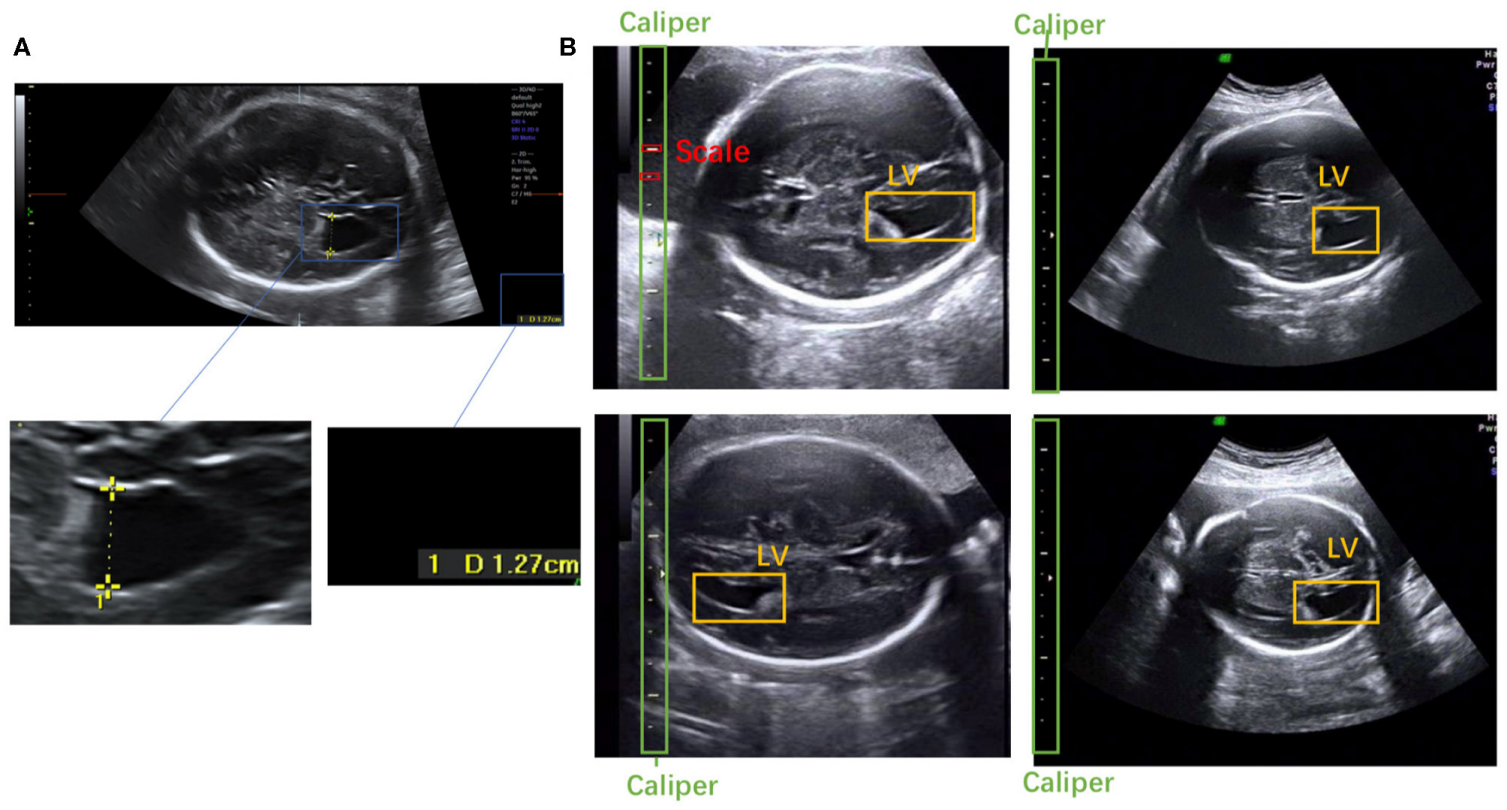
**(A)** Scanner-located LV width; the yellow crosses represent the endpoints, and the physical length of the LV is shown in the lower right corner. **(B)** Fetal LV images in different ultrasound instruments.

Ultrasound (US) is widely used in prenatal diagnosis because it is non-radiative, noninvasive, real-time, and inexpensive (1, 2). Ventriculomegaly, one of the most common abnormal findings in prenatal diagnosis, is often a sign of central nervous system malformation, chromosomal abnormalities, intrauterine infections, or other problems (3, 4). Ventriculomegaly can be diagnosed by measuring the fetal lateral ventricles (LVs) in standard plane images of the fetal brain. Currently, to measure the width of LVs, human scanners determine the maximum distance by marking two endpoints on the inner and outer edges of the LV. The line segment between these endpoints is considered the diameter of the LV and its length is generally referred to as the LV width (5), as shown in Figure 1A. However, such manual measurement requires extensive and comprehensive clinical knowledge of fetal LVs. It is a challenging task, especially for novice scanners. Additionally, scanners often suffer from repetitive stress injuries caused by multiple keystrokes (6). Therefore, it is necessary to develop automatic methods for fetal LV measurement, and crucial image-processing issues must be resolved to achieve a more accurate and efficient obstetric examination. Although the automatic measurement of fetal biometrics—such as head circumference (7, 8) and femur length (9, 10)—has attracted widespread attention in recent years, work on fetal LV measurement is rare.

To the best of our knowledge, this is the first study to propose an automatic measurement method for fetal LV width based on two-dimensional (2D) US images using deep learning. Several sample images of fetal LVs and the caliper in different US instruments are shown in Figure 1B.

**Figure 2:**
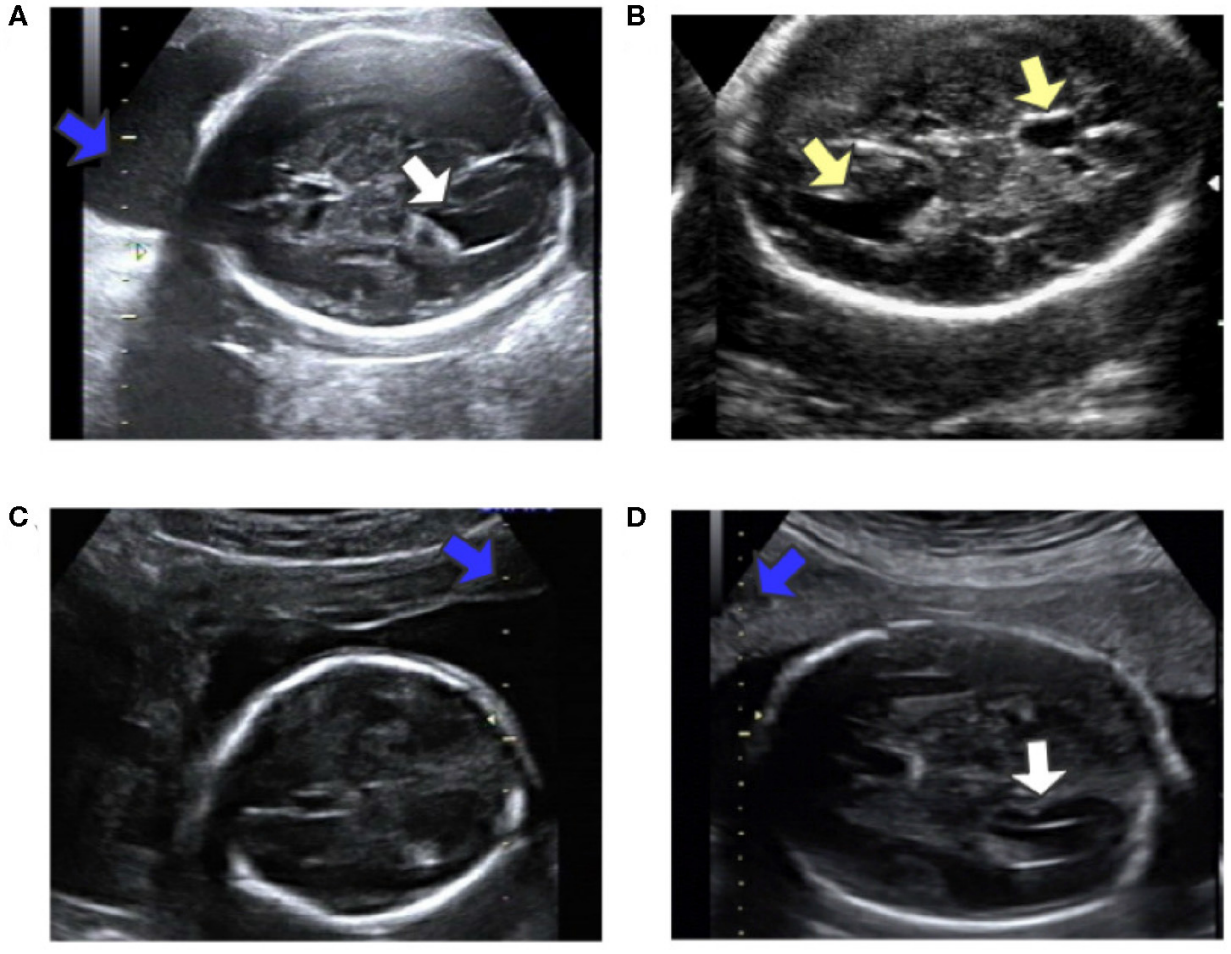
**(A)** Obvious fetal LV. **(B)** Images sometimes contain other similar structures. **(C)** Different location of the caliper than in **(A)**. **(D)** Fuzzy fetal LV, with a different type of caliper from that in **(A)**. Blue arrows indicate the caliper; yellow arrows indicate the LV and similar structures; white arrows indicate the LV.

Automatic measurement of fetal LV width remains a challenging task, as illustrated in Figure 2. The difficulties lie in three aspects: (1) The poor quality of the image can be an obstacle to accurate detection and segmentation of the fetal LV. For instance, the boundary of the LV may be blurred, as indicated by the white arrows in Figures 2A,D, which can result in a large overlap with adjacent tissues or anatomical structures, as highlighted by the yellow arrows in Figure 2B. (2) Because of differences between the location and type of calipers used, indicated by the blue arrows in Figures 2C,D, it is difficult to obtain the essential pixels-per-centimeter (PPC) parameter, which is the number of pixels in one centimeter of an image and is used to convert pixel length to physical length. (3) The subjectivity of manual measurements can cause issues, and the poor performance of the manual implementation is attributable to a lack of standardized training. Although standard definitions of the LV width are available, the widest part of the LV is determined by scanners manually marking two points on its inner and outer edges, as shown in Figure 1A.

To tackle these challenges, we develop a framework for automatic LV measurement based on deep learning. Specifically, we decompose the LV measurement task into three subtasks. First we train a Mask R-CNN (11) convolutional network on 2,400 images of fetal LV. The trained model can effectively learn and extract discriminative features from the training images and is able to perform joint classification, detection, and segmentation tasks simultaneously. Morphological operations (12) and prior knowledge are combined to enable extraction of the caliper scales in such a way that interference with other structures and tissues in the images is avoided. In the second step, we extract the caliper scales using prior knowledge. From these scales the PPC is calculated precisely. Then, we employ the minimum enclosing rectangle (MER) method to find the diameter and use the Euclidean distance to calculate its pixel length. Finally, the pixel length is transformed to the physical length of the LV by the PPC. The proposed method is evaluated on a self-collected dataset. Experimental results reported in **Table 4** demonstrate the superior performance of the method.

## 2. Materials and Methods

**Figure 3:**
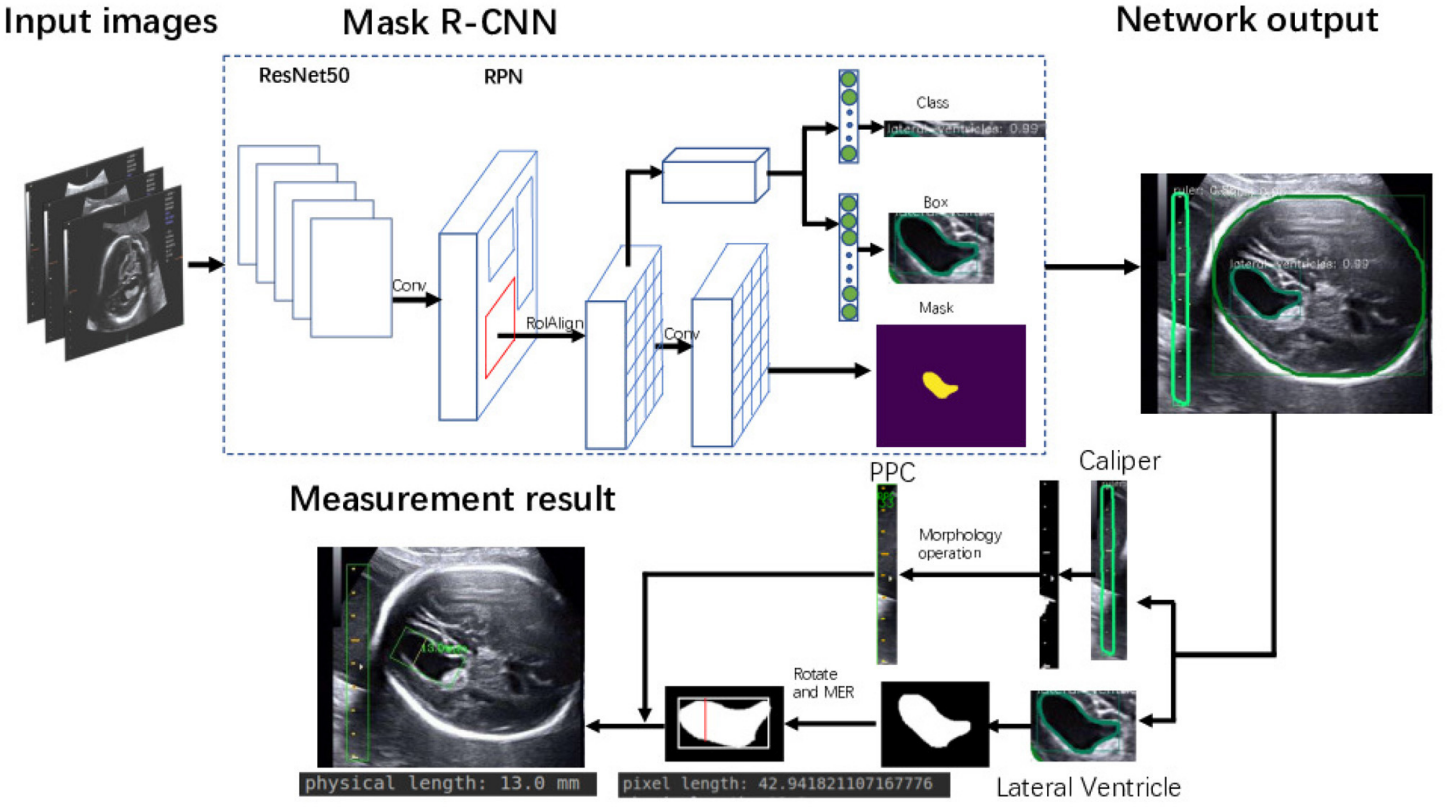
Flowchart of the proposed framework for automatic LV measurement.

The framework of the proposed method is summarized in Figure 3. Given heterogeneous sources of US images, Mask R-CNN can automatically detect and segment the caliper and the fetal LV simultaneously. Then, the PPC is obtained from the detected caliper using prior knowledge, and the pixel length of the LV is obtained by the MER method. Finally, the fetal LV width measurement is obtained by transforming the pixel length to a physical length using the PPC.

### 2.1. Image Acquisition

All examinations and diagnoses were carried out during routine screenings at the First Affiliated Hospital of Sun Yat-Sen University, China, from March 2010 to February 2018, by a team of 15 doctors with 3–22 years of experience. Images of fetal standard transventricular planes are required to assess fetal LVs, according to the guidelines of the International Society of Professionals in Ultrasound for Obstetrics and Gynecology (ISUOG). Ten different US machines provided by six different manufacturers (GE Voluson 730 Expert/E6/E8/E10, Aloka SSD-a10, Siemens Acuson S2000, Toshiba Xario 200 (Tus-X200), Samsung UGEO WS80A, and Philips EPIQ 7C) were utilized for data acquisition.

We acquired a total of 2,900 images, comprising 1,694 normal LVs, and 1,206 ventriculomegaly LVs, from the above US instruments, as well as 2,079 US images containing only calipers. To verify the robustness of the model, we collected 200 test images that contain neither LVs nor calipers to serve as negative samples. Our dataset is large enough to adequately represent the various LV images commonly seen in clinical practice. The ground truth of the LVs and calipers in these images were labeled by three experienced scanners. We randomly selected 2,400 images containing LVs and calipers and 1979 images containing only calipers to constitute the training dataset. The testing dataset is made up of 300 images containing LVs and calipers as positive samples and the 200 images without LVs and calipers as negative samples. The 1,979 images containing only calipers are used to improve the recognition of calipers.

### 2.2. The Mask R-CNN Model

The complex task of measuring the width of LVs can be disentangled into a few simple problems that are easily solved with convolutional neural networks (CNNs). We trained a Mask R-CNN to simultaneously perform two localization tasks and two segmentation tasks. In previous studies, deep learning has produced state-of-the-art results in many computer vision and medical image analysis problems, including prediction of protein function (13), classification of non-metastatic nasopharyngeal carcinomas (14), discovery of m6A sequences (15), image segmentation using new iterative tri-class thresholding techniques (16), qualitative assessment of fetal head US images (17), and detection of breast cancer (18). We choose to employ the deep learning algorithm Mask R-CNN, which combines object detection, object classification, and object segmentation. Notably, the excellent feature extraction ability of the deep learning network offers the potential of resolving the aforementioned issues in LV width measurement.

**Figure 4:**
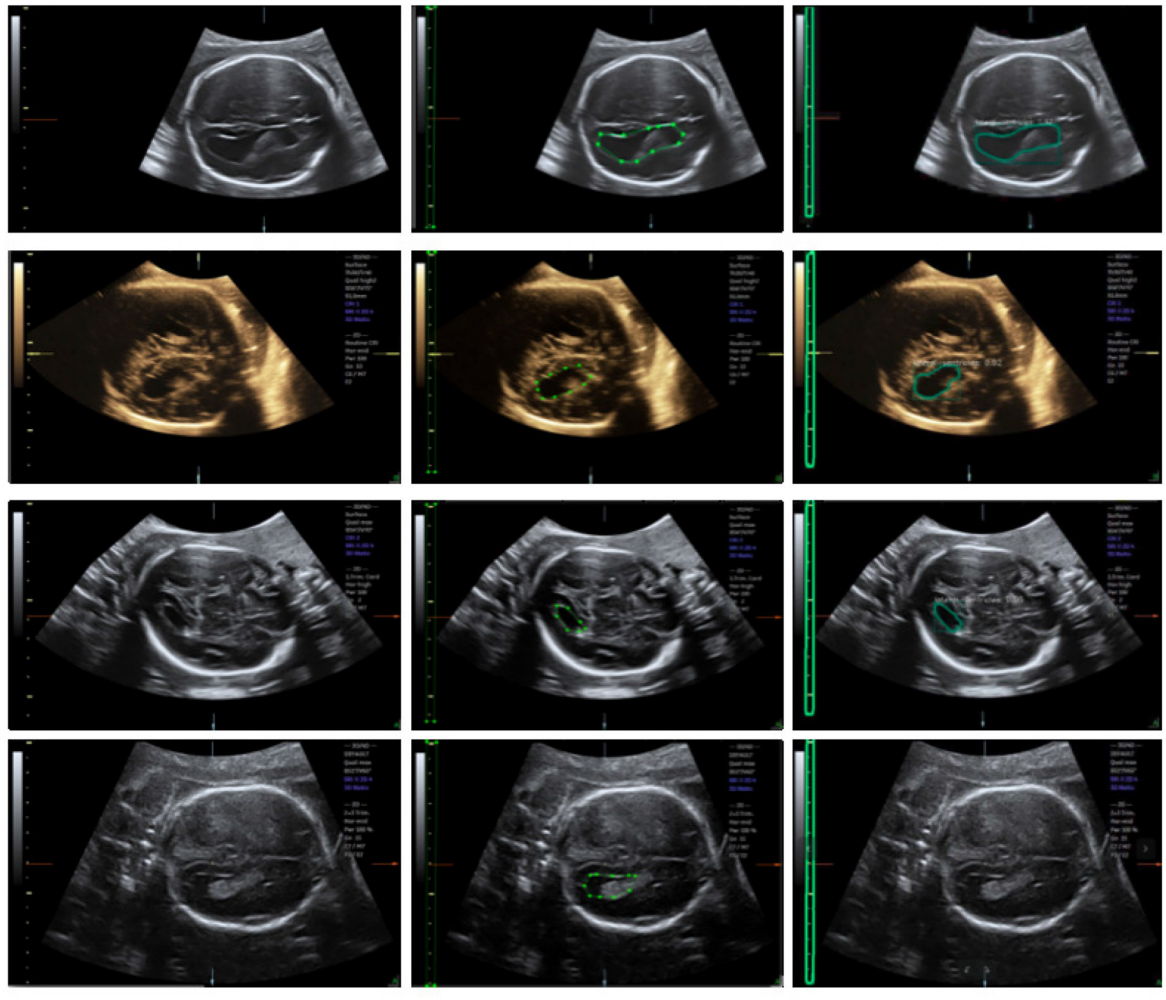
Ultrasound fetal brain image: from left to right the columns show the raw image, the ground truth labeled by the scanner, and the result detected by Mask R-CNN.

We trained an end-to-end 50-layer ResNet (19) with Feature Pyramid Networks (FPN) (20). Specifically, ResNet50 learns a set of image filters at multiple spatial scales and produces hierarchical feature maps of increasing coarseness. FPN combines the low-level features and high-level increase the receptive field and invariance. Then, the feature map generates many candidate region proposals through the Region Proposal Network (RPN). The class branch outputs the categories and confidence scores of the predicted anatomy. The bounding boxes of the target are obtained by the box branch. Subsequently, the mask branch learns to up-sample the coarse feature maps to produce a pixel-wise label prediction at the resolution of the input image. The hyperparameters are set as follows: the height and width of the input images are scaled down to 600 pixels; the training batch size is set to six images per batch; the initial learning rate and the number of iterations of the model are set to 0.02 and 20,000, respectively; and the remaining hyperparameters are set to their default values given in previous work (11). The Mask R-CNN network was trained with a training dataset, and the network output is shown in Figure 4. The network produces three results: bounding boxes, pixel-wise segmentation, and recognition confidence values for fetal LVs and calipers.

### 2.3. PPC Calculation With Morphological Operations and Prior Knowledge

Because of differences in the type, shape, and location of the caliper, it is difficult to obtain an accurate value for the PPC. To measure the LV width accurately, we incorporate clinical prior knowledge into our algorithm to guide the precise estimation of the PPC.

Through observation, two types of calipers are identified, which we refer to as the 10-caliper and the 5-caliper. For the former, the physical length between adjacent scales is 10 mm, as shown by the blue arrow in Figure 2A; the latter has a 5 mm distance between adjacent scales, as shown by the blue arrow in Figure 2D. The caliper scales are embedded in the ultrasonic structure, and the background noise poses an obstacle to extraction of the scale, as indicated by the blue arrow in Figure 2A. Obviously, the background and caliper scales cannot be distinguished directly via a threshold approach.

**Figure 5:**
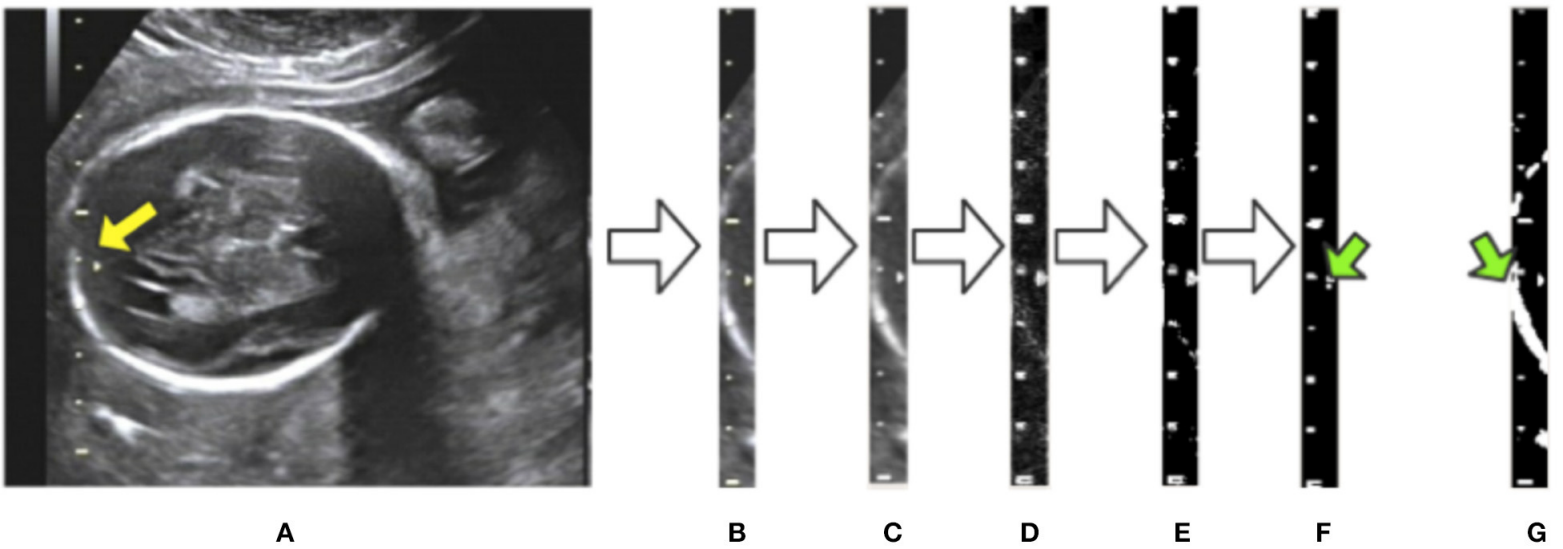
Visualization of background noise filtering: **(A)** raw image; **(B)** intercept of the caliper; **(C)** conversion to gray-scale; **(D)** edge detection by the Laplace operator; **(E)** binarization from gray-scale to monochrome; **(F)** morphological open operation. **(G)** The result of no morphological processing on the image. The green arrows indicate the difference between the treated image in **(F)** and the untreated one in **(G)**.

To eliminate the effects of background noise on scale extraction, we first crop the caliper from the raw image using the bounding box given by Mask R-CNN and process it separately. We then convert the caliper images into gray-scale. The Laplace operator is used for morphological operations after comparing with the experimental results. Large highlighted blocks of complex background are effectively discarded, as shown in Figure 5D. Next, we binarize the gray-scale image using a threshold value of 127 to obtain a monochrome image and then perform a morphological open operation on the monochrome image to eliminate a small amount of background noise. A visualization of these processing steps is shown in Figure 5. Figure 5G displays a binary image without the Laplace and open operations. As indicated by the green arrows in Figures 5F,G, the highlighted object is successfully filtered and the scales are preserved adequately after the Laplace and open operations. The background noise filtering is used preliminarily to filter out the part of the image that does not belong to the caliper.

**Figure 6:**
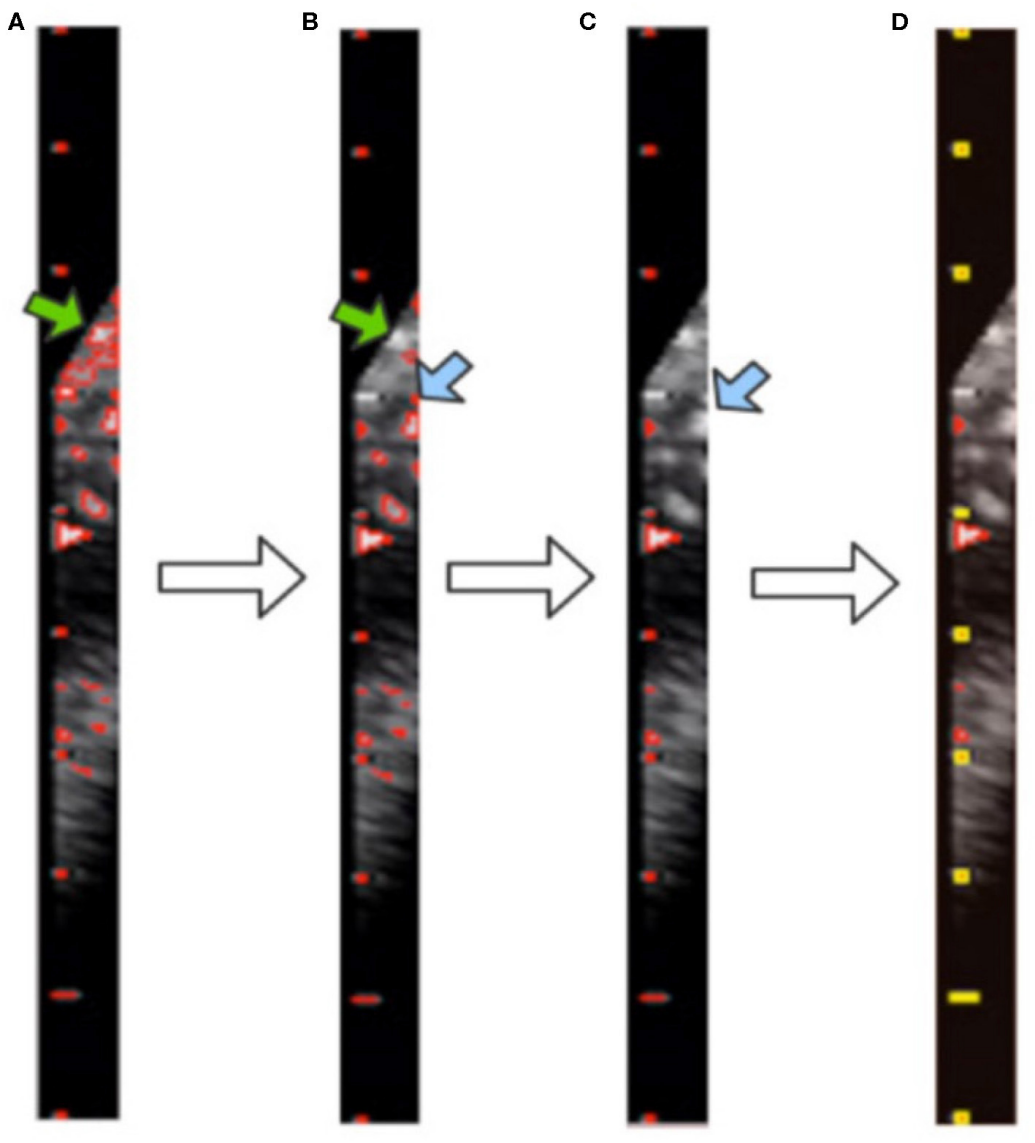
Visualization of contour filtering: **(A)** finding contours; **(B)** filtering out large contours; **(C)** filtering out edge contours; **(D)** filtering out inner contours. The yellow rectangles indicate the scales of the caliper found by our method.

**Figure 7:**
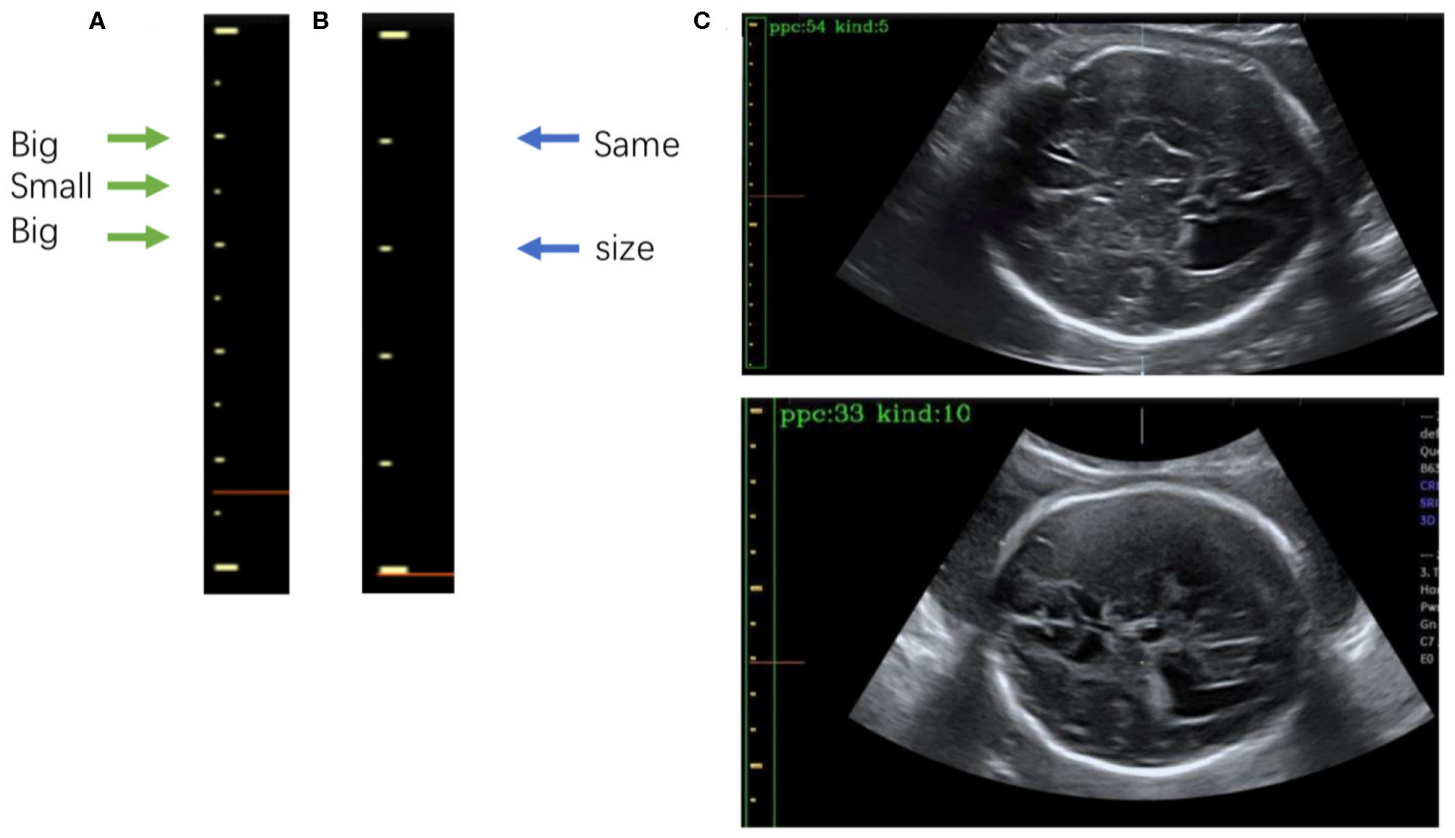
**(A)** The 5-caliper, with the green arrows indicating the “big-small-big” scale change law. **(B)** The 10-caliper, where the blue arrows indicate adjacent scales of the same size. **(C)** Results of caliper type judgments and PPC calculations; in each image the green box bounds the position of the scales given by Mask R-CNN, and the yellow box shows the caliper scales extracted by our method. The PPC value and caliper type are shown in the upper left corner of each image.

After morphological processing, the background noise is minimized and all contours in the image are obtained by using the “findContours” function of OpenCV, as shown in Figure 6A. These contours consist of pixel points (*x, y*). From our observations, the caliper scales follow three rules, as shown in Figure 7A. First, the scales belonging to a caliper contain a small number of pixel points. Second, the scales are all on the same *y*-axis. Third, the distances between adjacent scales are fixed.

Although the above step reduces the noise, it still cannot achieve the full denoising effect. To this end, we further propose contour filtering for taking into account prior knowledge of the scale. The aim of the following steps is to thoroughly eliminate noise, so as to identify the type of caliper and determine the PPC more accurately. First, we filter out the contours that have pixel numbers larger than the threshold value of 30, i.e., the large background contours that have not been cleaned by the morphological processing, indicated by the green arrows in Figures 6A,B. Next, we count the contours that intersect with each *y*-axis by traversing all *y*-axes in the caliper image. The *y*-axis with the greatest number of intersecting contours is regarded as the axis along which the scales are located. This step involves filtering out the small and medium contours of the image edge, as indicated by the blue arrows in Figures 6B,C. Then, the mode is obtained as the pixel distance between adjacent scales via calculating and recording the distance between adjacent contours. The position of each contour is represented by the point at the upper left corner of the contour. Finally, all scale contours are obtained via the pixel distance, and the “minAreaRect” function of OpenCV is utilized to generate a rectangular bounding box for each scale contour, to filter out the small contours between scales. After these steps have been applied, the remaining contours are considered the scales of the caliper. This process is illustrated in Figure 6D, where the yellow rectangles indicate the scales of the caliper obtained by our method.

After extracting the scales, we calculate the PPC. The two kinds of calipers have their unique rules of change in scale size: the 5-caliper has a “big-small-big” change rule, as shown in Figure 7A, whereas the 10-caliper has adjacent scales of the same size, as shown in Figure 7B. The type of caliper is determined by these rules and the scales extracted using the above procedure. In the case of the 5-caliper, the PPC of the image is the distance between adjacent scales multiplied by 2; for the 10-caliper, the PPC is the distance between adjacent scales. The caliper type and PPC results are shown in Figure 7C at the upper left corner of the image. The green box surrounds the position of the scales obtained by Mask R-CNN, and the yellow box indicates the caliper scale extracted by our method.

### 2.4. Locating LV Diameter and LV Measurement

Clinically, the widest location of the LV is determined by scanners, who mark two points on the inner and outer edges of the LV. To find the widest location of the LV in images, the MER method is used to simulate the judgment of scanners.

**Figure 8:**
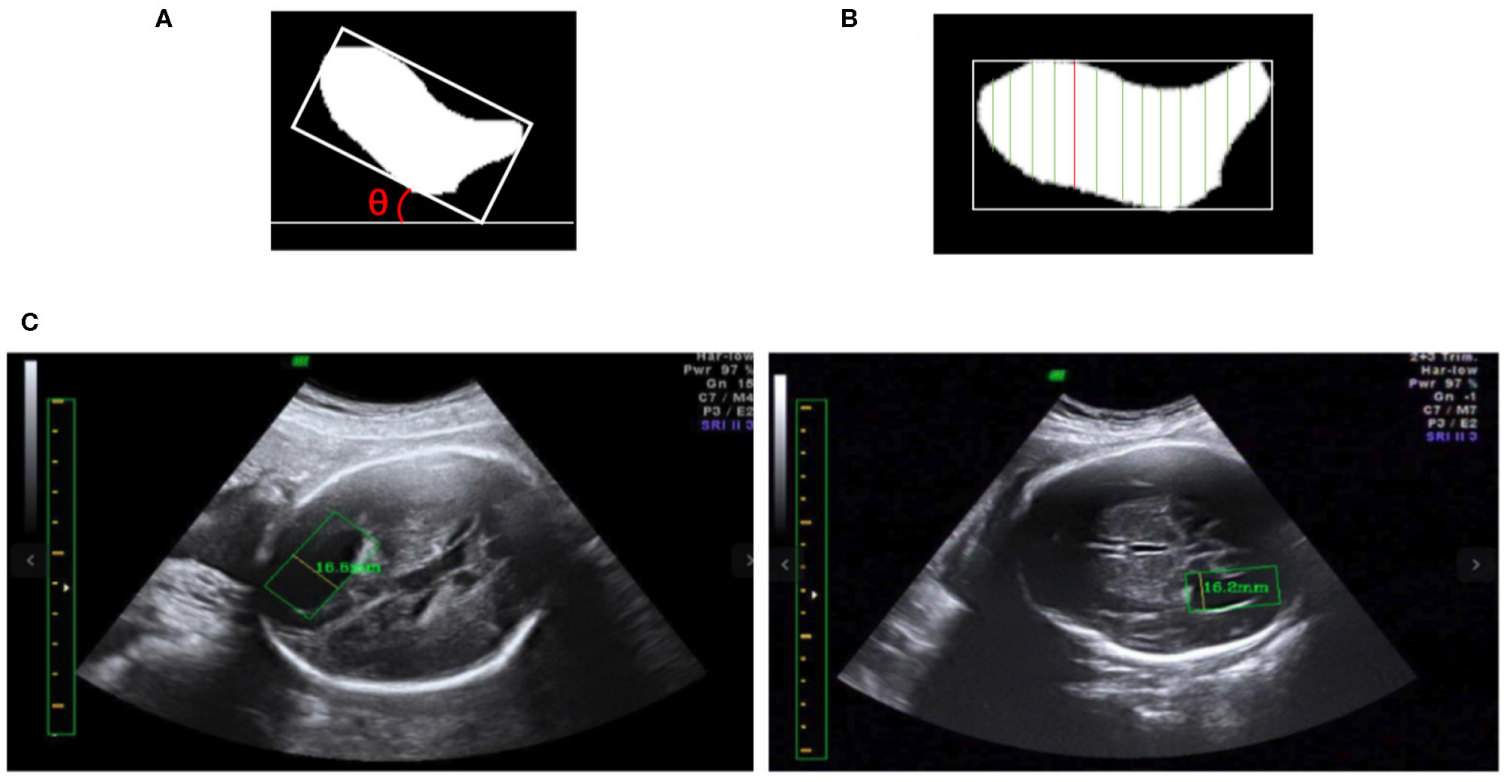
**(A)** The minimum angle θ between the *x*-axis and the enclosing rectangle of the LV image is determined. **(B)** After rotating the image to make it horizontal, the vertical green lines are candidate diameters; the red line is the longest diameter. **(C)** Two LV measurements obtained using our method; in each image the yellow line inside the green box is the diameter of the LV as determined by our method, and the measurement results are shown near the yellow line.

We obtain the angle θ between the *x*-axis and the edge of the enclosing rectangle with the smallest angle to the *x*-axis, as illustrated in Figure 8A. First, the image is rotated by angle θ in the opposite direction to give a horizontal LV, as shown in Figures 8A,B. Then, we traverse all *y*-axes and obtain two points intersecting the contour of the LV; see the green lines in Figure 8B. We calculate the pixel distance between these two points using the Euclidean distance. The largest pixel distance is regarded as the width of the LV, and the diameter of the LV is obtained as the line connecting the two points, i.e., the red line in Figure 8B. Finally, the physical length of the LV diameter is calculated as the pixel length divided by the PPC. Figure 8C shows a visualization of the results.

## 3. Results and Discussion

We conduct three experiments to assess the reliability and efficacy of the proposed framework for automatic fetal LV measurement: (1) To evaluate our method's detection and segmentation ability for LVs and calipers, we test the trained network model with a testing dataset containing 500 images. (2) We assess the accuracy of the PPC acquisition method. (3) We compare the measurement errors between the LV width measured by our method and the ground truth measured by three scanners.

Our data were labeled by three experienced scanners. The diagnosis results and measurements produced by their consensus judgments serve as the ground truth in Table 1. The physical lengths of the LVs in **Table 4** were also measured by doctors.

**Table 1:** Caliper and LV prediction results.

**Caliper**		**Predicted result**		**Total**
		**Yes**	**No**	
Ground truth	Yes	290	7	297
	No	0	203	203
	Total	290	210	500
Precision	Recall	Sensitivity	Specificity	Accuracy
100%	97.64%	100%	96.67%	98.6%
**LV**		**Predicted result**		**Total**
		**Yes**	**No**	
Ground truth	Yes	280	15	295
	No	5	200	205
	Total	285	215	500
Precision	Recall	Sensitivity	Specificity	Accuracy
98.25%	94.91%	98.25%	93.02%	96%

### 3.1. Detection and Segmentation Ability of Mask R-CNN

As shown in Table 1, to evaluate the proposed method's ability to recognize LVs and calipers, we record its precision, recall, sensitivity, specificity, and accuracy on the test set.

The results indicate that the trained Mask R-CNN identifies the LV and the caliper with accuracies of 96 and 98.6%, respectively. The precision of the model is 100% for calipers and 98.25% for LVs and indicates that the positive samples identified by the network have high confidence. Our model performs well for negative samples, with a specificity of 96.67% for calipers and 93.02% for LVs.

**Figure 9:**
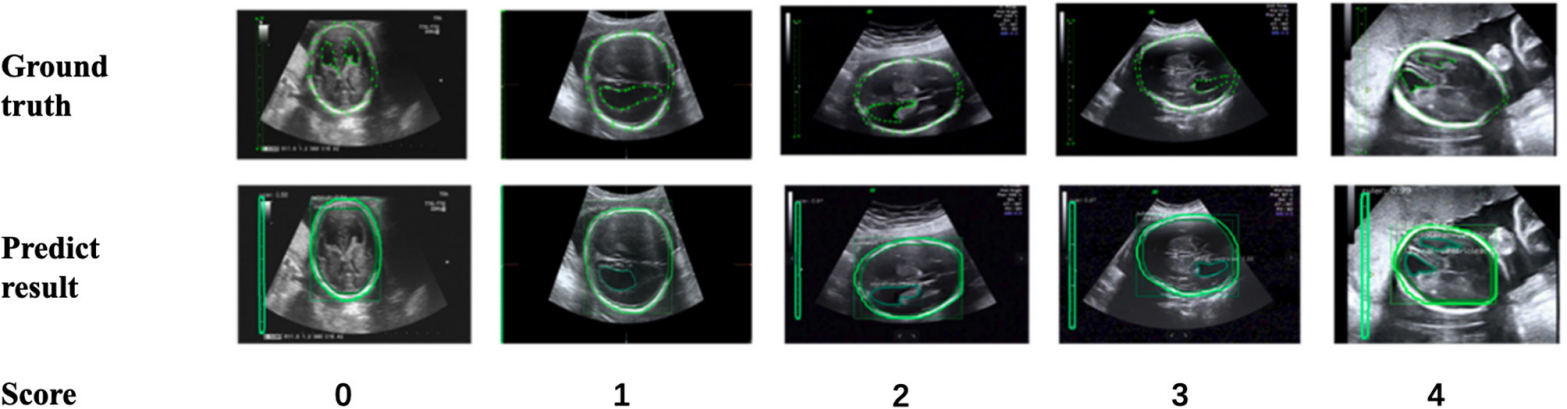
Scoring criteria of three ultrasound experts, who scored the degree of fitting between predicted LV contours and the ground truth on a scale from 0 to 4.

In addition, to verify the accuracy of the LV contour segments obtained by Mask R-CNN, we invited three US experts to score the degree of fitting between the predicted LV contours and the ground truth on a scale from 0 to 4. The scoring criteria are shown in Figure 9, and the results are reported in Table 2. Predicted results scoring 4 points accounted for 77.4% of the cases, with an average score of 3.5. To summarize, the network appears to accurately predict the LV contours.

**Table 2:** Expert scoring results.

**Score**	**Number**	**Percentage (%)**	**Average**
0	15	5	
1	10	3.3	
2	15	5	
3	28	9.3	
4	232	77.5	
Total	300	100	3.5

**Figure 10:**
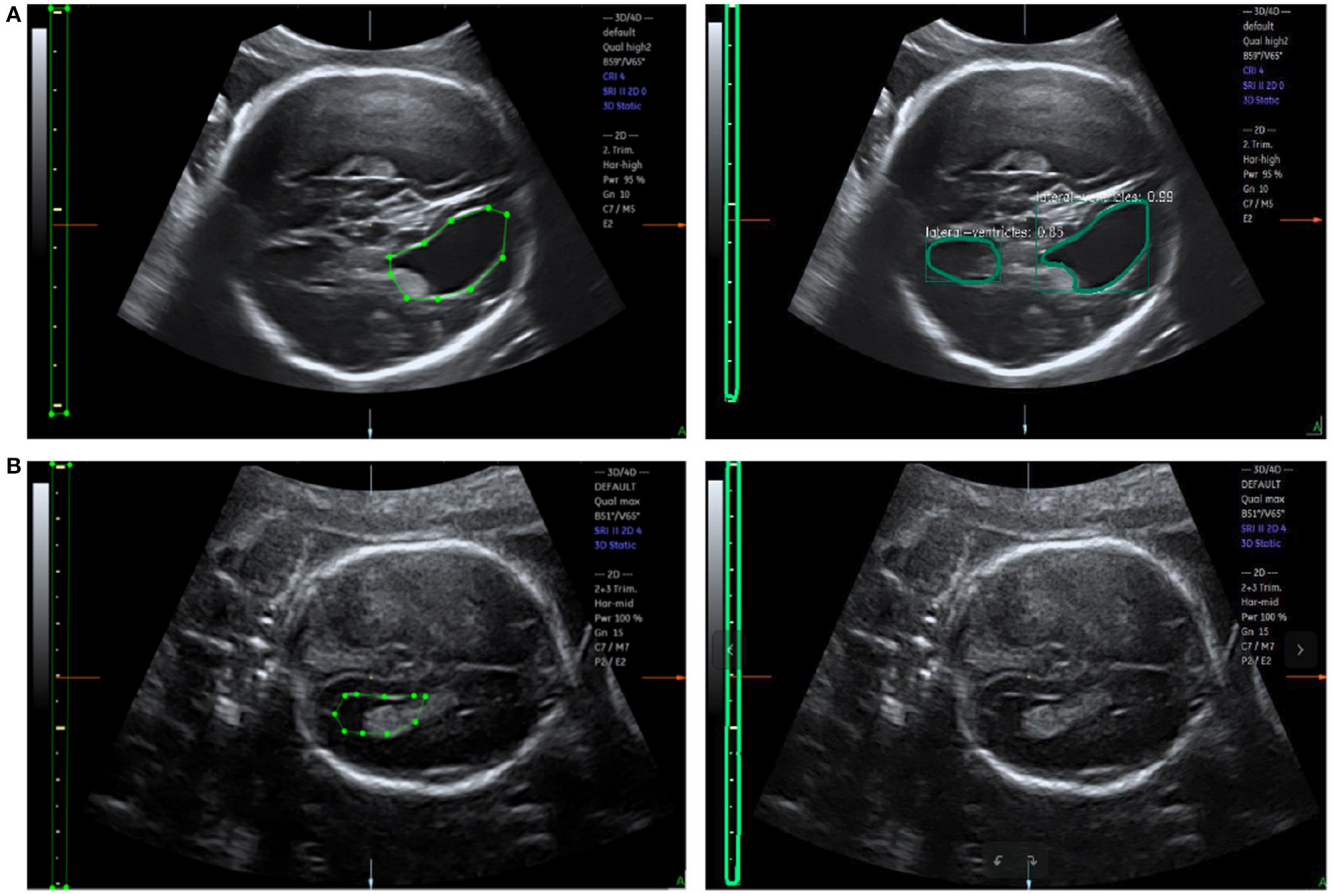
**(A)** Error recognition and **(B)** failure recognition for LVs. The first row shows the ground truth and error recognition; the second row shows the ground truth and failure recognition.

The accuracy of LV detection is lower than that of caliper detection. A separate analysis of incorrect recognition cases, as shown in Figure 10A, indicates that structures similar to LVs are likely to be present in the images, demonstrating that object detection in US images is still a challenging task. As can be seen in Figure 10B, the unrecognized images are too dark, and the contour of the LV is not obvious. A possible reason is that the training dataset is insufficient. In future work, more LV images will be collected and used for optimization of the network structure to improve the detection of LVs.

Under the same hardware conditions (CPU Intel Core i7-7700 3.60 GHz X8; GPU GeForce GTX 1060 6 GB / PCIe / SSE2), we train DeepLab V3+ networks with the same training set. The hyperparameters are chosen as follows: train_crop_size is set to 401 × 801, train_batch_size is set to 2, and the model is iterated 40,000 times; the remaining hyperparameters are set to their default values in previous work (21). Figure S1 shows the comparison results. The experimental results indicate that the performance is worse than that of Mask R-CNN, a possible reason being that the images in the training set are relatively small, only 512 × 512. In contrast, the images in our training set are large enough, with most of the sizes being 700 × 1,400.

### 3.2. Accuracy of the PPC

The accuracy of the obtained PPC depends on the judgment of the caliper type. To better represent the accuracy of caliper type judgments with different data complexities, we randomly select 20 images with black backgrounds and 80 images with complex backgrounds to make up a 100-image test dataset. Table 3 demonstrates the accuracy of our method in caliper classification.

**Table 3:** Accuracy of caliper type judgments.

	**Number**	**Correct**	**Error**	**Accuracy (%)**
Total	100	92	8	92
Black	20	19	1	95
Complex	80	73	7	91.25

The experimental results indicate that our method performs well in the recognition of caliper types against a black background, with an accuracy of 95%; however, the accuracy of recognition of caliper types against a complex background could be further improved.

### 3.3. Measurement Error

**Figure 11:**
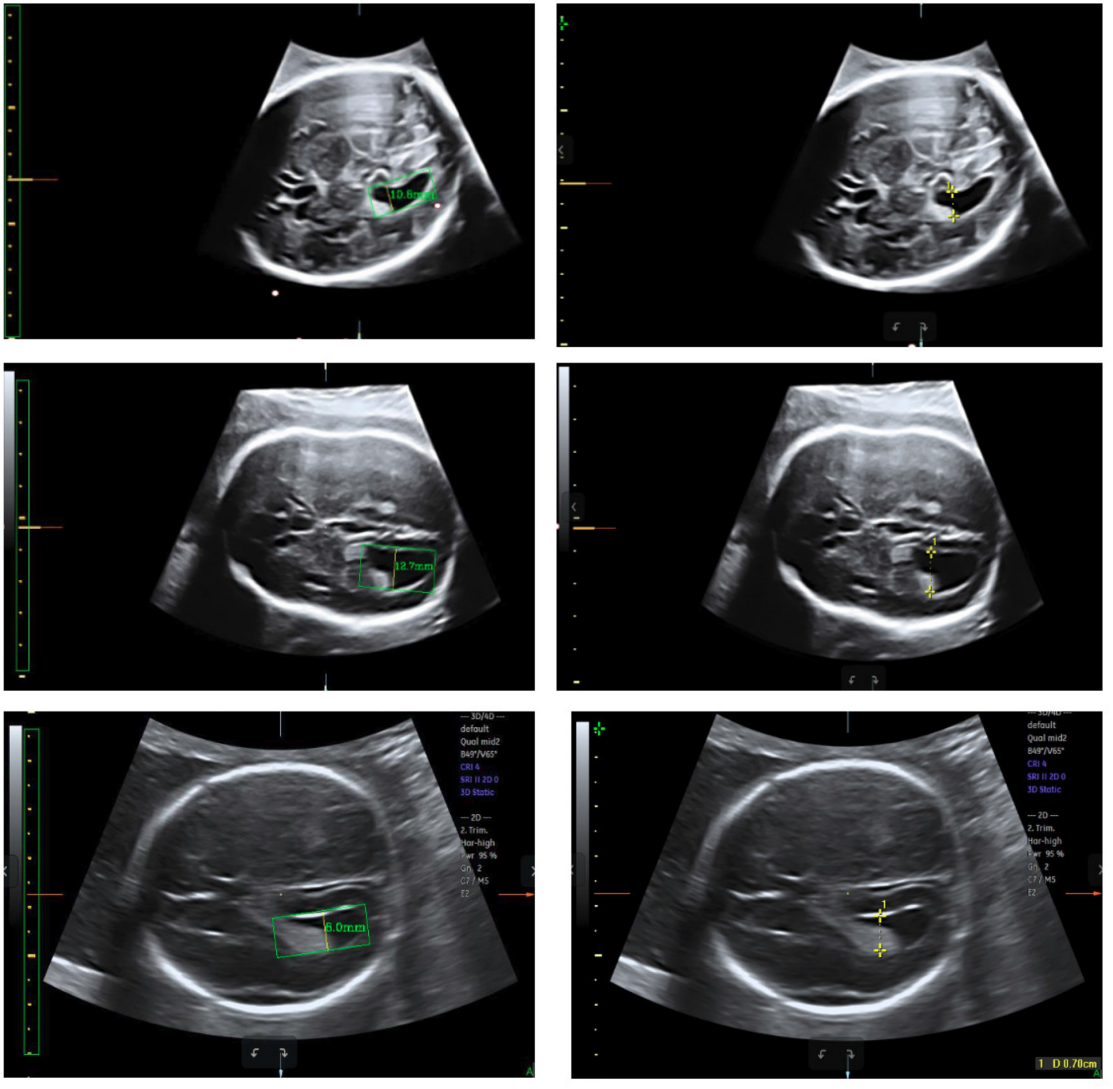
Measurement results obtained using our method (left) compared with the ground truth (right).

Test sets consisting of 200 LV images measured by scanners are used as the ground truth. We measure the same LVs using our method and compare the results. Notably, the LVs are not recognized in 19 of the 200 images; these images are considered to be 0 mm in the measurement error statistics. Figure 11 displays LV measurement results obtained by our method, compared with the LV ground truth provided by experts. The LV diameters determined by our method are quite close to the ground truth, and the measurement error is small. The mean absolute error, standard deviation, root mean squared error, and time consumption of our method are listed in Table 4. Because 19 of 200 images are considered to be 0 mm, the standard deviation is large, 3.4 mm. The experimental results demonstrate that our method is accurate and efficient for measuring fetal LV width; for example, the time consumption is 0.13 s per image and the mean absolute measurement error is 1.8 mm.

**Table 4:** Quantification of the performance of the proposed method in terms of mean absolute error (MAE), standard deviation (SD), root mean squared error (RMSE), and average time consumed (ATC).

**MAE (mm)**	**Percentage of MAE**	**SD (mm)**	**RMSE (mm)**	**ATC (s)**
1.8	18.92%	3.4	2.38	0.13

## 4. Conclusion

This paper describes an automatic method for measuring the width of LVs in 2D US images. To the best of our knowledge, this is the first study proposing an automatic measurement method for fetal LVs based on 2D US images using deep learning. Our method is able to automatically recognize and locate the fetal LV in 2D US images and can measure the width of the LV rapidly and accurately. Moreover, our model, with slight modifications, can be extended to the measurement of other fetal biometrics, such as femur length and head circumference. The demonstrated robustness of the model implies that it is also a promising tool to be used with various ultrasonic instruments to facilitate quick clinical prenatal diagnosis. The experimental results on 200 LV images indicate that the performance of our proposed method is close to the manual method of LV measurement in terms of accuracy and efficiency.

The measurement errors of our method mainly arise from three sources: inadequate fitting of the LV contour, inaccurate PPC calculation, and inaccurate diameter location. In future work, we will focus on reducing measurement errors by using a greater amount of LV data, improving the network structure to enhance its abilities of detection and segmentation, and modifying the location algorithm for determining LV diameter. Our long-term goal is to develop an automatic system that can measure all biometrics based on fetal US images.

## Data Availability Statement

The datasets for this article are not publicly available because ownership of data sets belongs to cooperative hospitals. Requests to access the datasets should be directed to Hongmin Cai, hmcai@scut.edu.cn.

## Ethics Statement

The study protocol was approved by the Institutional Review Board of the First Affiliated Hospital of Sun Yat-sen University.

## Author Contributions

XC wrote the majority of the manuscript. TD carried out the supplementary experiments and responded to the comments. MH, NW, and ML labeled all the data. LZ and HX checked the data. JX performed the experiments. HC checked the manuscript. All authors contributed to manuscript revision.

## Conflict of Interest

NW and JX were employed by company Guangzhou Aiyunji Information Technology Co., Ltd., Guangzhou, China. The remaining authors declare that the research was conducted in the absence of any commercial or financial relationships that could be construed as a potential conflict of interest.
