# Supplementary material for: Automatic Measurements of Fetal Lateral Ventricles in 2D Ultrasound Images Using Deep Learning
Source: Front Neurol. 2020 Jul 17;11:526. doi: 10.3389/fneur.2020.00526 (PMC7380113; doi:10.3389/fneur.2020.00526)
Supplement: Supplementary file 1 [file Data_Sheet_1.PDF]

Supplementary Material

1 SUPPLEMENTARY TABLES AND FIGURES

For more information on Supplementary Material and for details on the different file types accepted, please see the Supplementary Material section of the Author Guidelines.

Figures, tables, and images will be published under a Creative Commons CC-BY licence and permission must be obtained for use of copyrighted material from other sources (including re-published/adapted/modified/partial figures and images from the internet). It is the responsibility of the authors to acquire the licenses, to follow any citation instructions requested by third-party rights holders, and cover any supplementary charges.

1.1 Figures

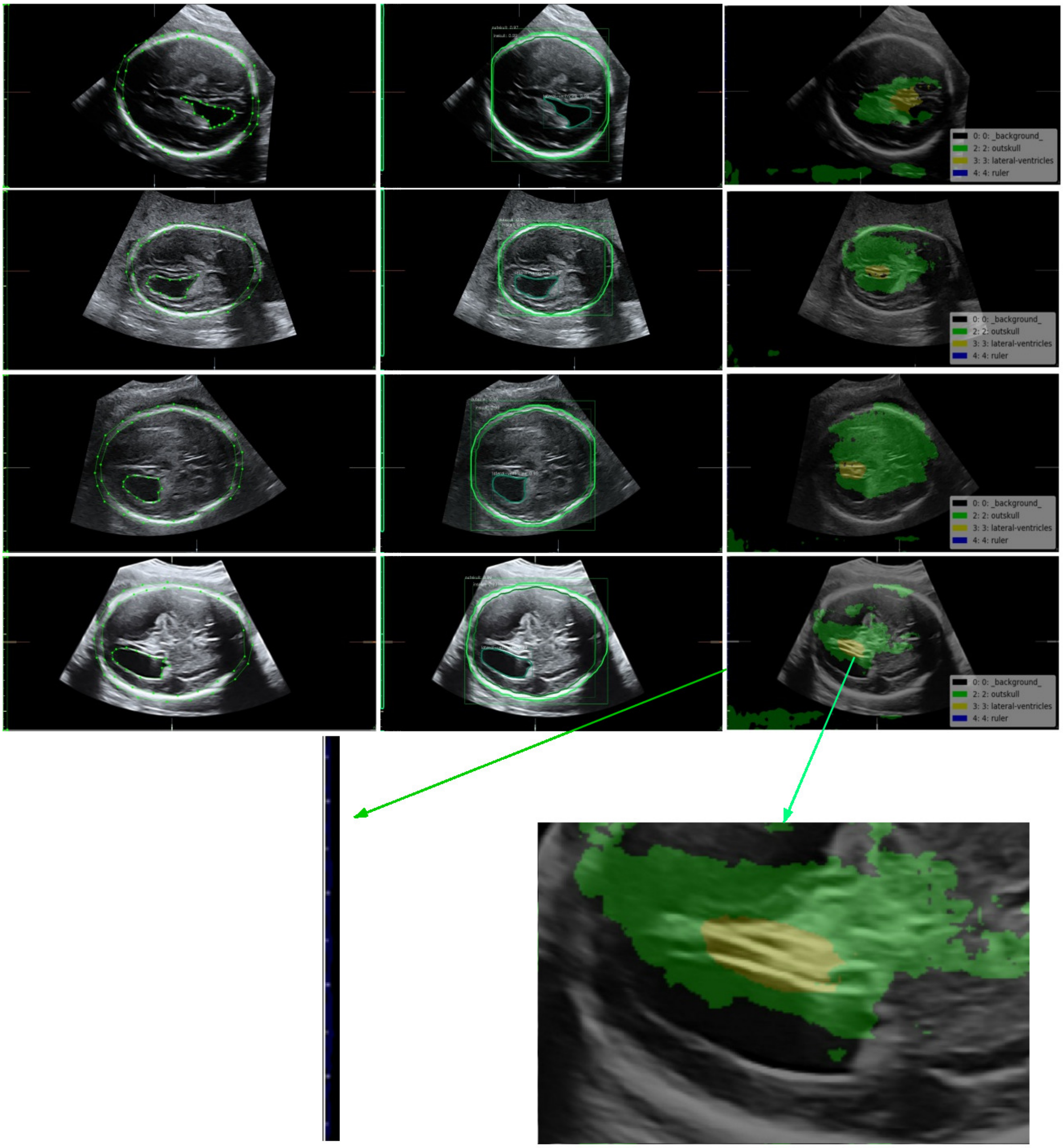

Figure S1. The left column is the ground truth. The center column is the result of Mask R-CNN. The right column is the result of DeepLab V3+.

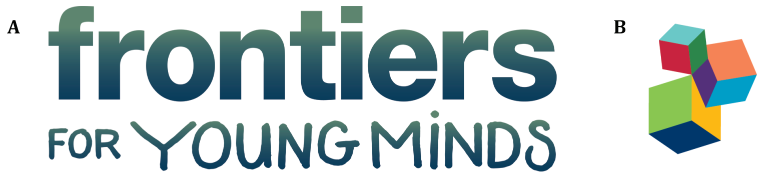

Figure S2. This is a figure with sub figures, **(A)** is one logo, **(B)** is a different logo.
